# Effectiveness of Manual Therapy, Customised Foot Orthoses and Combined Therapy in the Management of Plantar Fasciitis—A RCT

**DOI:** 10.3390/sports7060128

**Published:** 2019-05-28

**Authors:** Casper Grim, Ruth Kramer, Martin Engelhardt, Swen Malte John, Thilo Hotfiel, Matthias Wilhelm Hoppe

**Affiliations:** 1 Department of Orthopaedic, Trauma, Hand and Neuro Surgery, Klinikum Osnabrueck GmbH, 49076 Osnabrueck, Germany; martin.engelhardt@klinikum-os.de (M.E.); thilo.hotfiel@klinikum-os.de (T.H.); matthias.hoppe@klinikum-os.de (M.W.H.); 2 Physiopraxis Kramer, 49492 Westerkappeln, Germany; ruth@physiopraxis-kramer.de; 3 Department of Dermatology, Environmental Medicine and Health Theory, University of Osnabrueck, 49076 Osnabrueck, Germany; sjohn@uos.de; 4 Department of Orthopedic Surgery, Friedrich-Alexander-University Erlangen-Nuremberg, 91054 Erlangen, Germany; 5 Department of Movement and Training Science, University of Wuppertal, 42119 Wuppertal, Germany

**Keywords:** plantar fasciitis, heel pain, manual therapy, joint mobilization, customised orthoses, insoles, back pain

## Abstract

Background: Plantar fasciitis (PF) is one of the most common causes of plantar heel pain. Objective: To evaluate the effectiveness of three different treatment approaches in the management of PF. Methods: Sixty-three patients (44 female, 19 men; 48.4 ± 9.8 years) were randomly assigned into a manual therapy (MT), customised foot orthosis (FO) and a combined therapy (combined) group. The primary outcomes of pain and function were evaluated using the American Orthopaedic Foot and Ankle Society-Ankle Hindfoot Scale (AOFAS-AHS) and the patient reported outcome measure (PROM) Foot Pain and Function Scale (FPFS). Data were evaluated at baseline (T0) and at follow-up sessions after 1 month, 2 months and 3 months (T1–T3). Results: All three treatments showed statistically significant (*p* < 0.01) improvements in both scales from T0 to T1. However, the MT group showed greater improvements than both other groups (*p* < 0.01). Conclusion: Manual therapy, customised foot orthoses and combined treatments of PF all reduced pain and function, with the greatest benefits shown by isolated manual therapy.

## 1. Introduction

Plantar fasciitis (PF) is reported as the most common cause of plantar heel pain and is referred to as plantar fasciosis or fasciopathy, because these terms more accurately describe the inflammatory degenerative nature of the disease [1,2,3,4,5]. The prevalence rate ranges from 4% in general to 7% in older populations, and from 8% in athletes to 25% in runners [6]. In non-athletes, women are more frequently affected [1,7,8] and have a higher risk of persisting symptoms [9] than men.

The aetiology is largely unknown [2] and risk factors remain unclear. Obesity, prolonged standing, running, limited ankle dorsiflexion, shortened triceps surae, hindfoot malalignment and increased age are all considered as potential risk factors [2,4,6,10,11]; however, their scientific evidence is weak. The high occurrence rate and level of impairment require a better understanding not only of the diagnosis, but also of evidence-based recommendations for the therapy. Concerning the latter, the quality of the studies is heterogeneous and often several forms of therapy are carried out simultaneously [1,2,6]. This makes it difficult to determine the effectiveness of any individual therapy or to rank therapies in order of their effectiveness [4]. Many patients report having persisting or recurrent pain following treatment [12]. In addition to night splints, resistance training, corticoid injections and extracorporeal shockwave therapy, manual therapy and foot orthoses are commonly recommended interventions [13,14].

Due to limited research, there are however no clear arguments for the use of foot orthoses. The theoretical underpinning for their use includes improvement of the hindfoot alignment, the relief in plantar pressure to the origin of the PF and modification in heel pitch, which may alter the mechanical loading of the plantar fascia [15]. A lack of high quality evidence was found for the use of foot orthoses [6,16]. Statistically significant differences were not found between customised or prefabricated foot orthoses or soft and firm foot orthotic materials [2,6,13,17]. A longer duration of foot orthoses use was associated with impairment in the plantar fascia and toe flexor muscle function [12]. There are no two studies that used the same type of orthoses, which limits the comparisons between studies and suggestions as to which orthoses features may be most effective. What is considered most important is whether foot orthoses are beneficial to patients by effectively alleviating their symptoms [6].

There is only weak [1] or moderate evidence for short-term treatment [18,19] using manual therapy interventions. However, compared to physical therapy, patients needed fewer sessions, thereby reducing treatment costs [20]. Stretching of the calf muscles and improvement of the ankle dorsiflexion is often recommended [13]; however, this additional mobilisation was no more effective than stretching and ultrasound treatment alone [21]. In a study comparing customised foot orthoses versus mobilisation of the foot and stretching, the mobilisation group had better results after two weeks, but not after one or two months [17]. In only two of the reviewed studies [7,22], a single treatment was applied in isolation in the experimental group. The other two well-rated studies reporting significant improvements [1,23] used multiple interventions simultaneously. Thus, the studies did not allow conclusions to be drawn with regards to the effectiveness of manual therapy alone [4]. In an osteopathic study, the overall results were not significantly improved with three treatments [24].

After receiving conservative treatments, nearly 50% of patients still had symptoms when interviewed after nine years [9,25]. Patients with plantar heel pain had a high prevalence of lumbar back pain. Compared to the control group, more than twice as many patients with plantar heel pain had lumbar back pain with the corresponding risk being five times higher. Treatment of local and proximal restrictions, including those associated with back pain, may be justified for improving the management of PF [25]. Hence, in the current study, the spine was also evaluated and treated. To date, there has been no prospective, randomised, controlled trial in which manual therapy and customised foot orthoses were investigated in relation to back pain in patients with PF.

Thus, the aim of this study was to compare the effectiveness of manual therapy, customised foot orthoses and combined therapy in the management of PF.

## 2. Materials and Methods

The Ethics Committee of the University of Osnabrueck approved and accepted all procedures involved in this study (4/7/1043.5). The patients were consecutively recruited over a 36-month period. Patients were screened for eligibility by a foot and ankle surgeon. Inclusion criteria were: a clinical diagnosis of PF with symptoms for <6 months and an age ≥18 years. Exclusion criteria were: red flags for manual therapy interventions, previous surgeries, fractures, rheumatoid diseases, tumours and other forms of therapy during the study. All patients were informed about the procedures involved in the study and signed an informed consent form. Based on order of appearance, the patients were randomly assigned into one of the three groups: (i) manual therapy (MT) group, (ii) customised foot orthosis (FO) group and (iii) combined therapy (combined) group. The patients were then referred to a manual therapist, an orthopaedic technician or to both, respectively.

### 2.1. Examination Procedures

**Figure 1:**
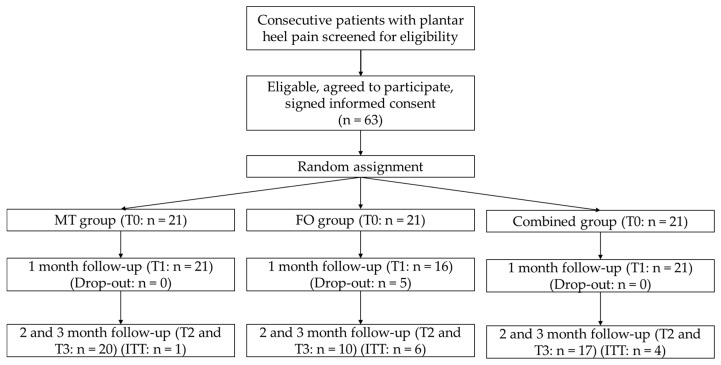
Flow-diagram of patient recruitment. Abbreviations: MT, manual therapy; FO, foot orthoses; ITT, Intention to treat; T0, baseline; T1–T3, follow-up sessions after 1–3 months.

All patients provided demographic information, medical history and previous treatments of PF. They received a physical examination at baseline (T0) and at follow-up sessions after 1 month, 2 months and 3 months (T1–T3). The primary outcomes of pain and function were evaluated using the American Orthopaedic Foot and Ankle Society-Ankle Hindfoot Scale (AOFAS-AHS) and the patient reported outcome measure (PROM) Foot Pain and Function Scale (FPFS). An intention to treat analysis was carried out using missing data from the last available value for the final evaluation [26,27]. Figure 1 shows the patient recruitment and sample sizes, drop-outs and intention to treat of the three groups.

### 2.2. Outcome Measures

The AOFAS-AHS includes both subjective, patient reported items in pain and function (60%) and objective, physician assessed items in function (40%). The AHS is scored from 0 to 100, where higher values indicate a better outcome [28,29]. The AHS was preferred over the commonly used Short Form 36 (SF-36), because the SF-36 has not been specifically studied in relation to foot and ankle disorders [4]. Additionally, the time required for the evaluation of the nine items of the AHS is lower than for the SF-36, which increases patient compliance in reporting data. Despite methodological criticisms, the AHS is an established and frequently used rating system, making it possible to compare the results with other studies [30,31]. The degrees of correlation and reliability provided an acceptable validity for the subjective scores; however, the reliability of the objective component of the AHS has yet to be reported [29,32]. There have been no reliable data published regarding the minimal clinically important difference (MCID) related to the AOFAS score [29]. The MCID of the AHS in hallux valgus surgery were indicated between 7.9 and 30.2, effect size derived 8.4 [33] or 8.9 out of 100 [34]. In the current study, the MCID was set at 10 out of 100. In the AHS, the subscale pain is one single item. Pain is however subjective, with PROM providing the most valid measure of the experience [35].

In order to obtain more differentiated values for the typical pain of PF, the Foot Pain and Function Scale (FPFS) was created with an 11-point numeric rating scale (NRS) from 0 to 10, where higher values indicate better outcomes. The low gradation of the NRS for pain compensates for the large variance in point values in the Ankle Hindfoot Scale [29]. The FPFS contains five questions on pain (first steps, during rest, on pressure, while standing, weight bearing) and five questions about function (limping, weakness, stiffness, restrictions in sports, at work). The highest possible total score is 100. The FPFS uses 20 questions from the Visual Analogue Scale Foot and Ankle (VAS FA) [31], all of which were validated against the SF-36 and the Hannover Questionnaire. VAS and NRS have a well-documented reliability and validity in a variety of populations [1,4]. Due to the heterogeneity between study results, no meaningful overall value for the MCID change can be determined. In the subgroup pain, the NRS median for the MCID was 15% [36], those considered as clinically important or “improved” ≥20%, clinically very important or ”much improved” ≥30% and ”very much improved” ≥40%, respectively [37,38,39].

Blinding is barely achievable with the application of manual therapy in interventional studies, making an even higher quality of the evaluation difficult [4]. Therefore, in the current study, assignment of the patients to treatment groups was blinded for the therapist. The form of intervention itself was recognisable to patients and therapists, though patients did not know if they were participating in an intervention or control group.

### 2.3. Interventions

The patients were treated with manual therapy twice during the first week and subsequently once per week for the remaining three-month period.

Patients in the manual therapy and combined group were evaluated with a standardised clinical examination. The therapist used pre- and post-tests for each joint of the foot and intervertebral segment of the spine. The order and type of treatment in therapy was standardised. The tests and joint mobilisations were performed talocrural for dorsiflexion, subtalar for eversion and inversion, and then tarsi transversal for pro- and supination. The sacroiliac joints and the symphysis pubica were assessed and mobilised as well as the intervertebral joints in the supine position, partially in lateral decubitus with rotation.

**Figure 2:**
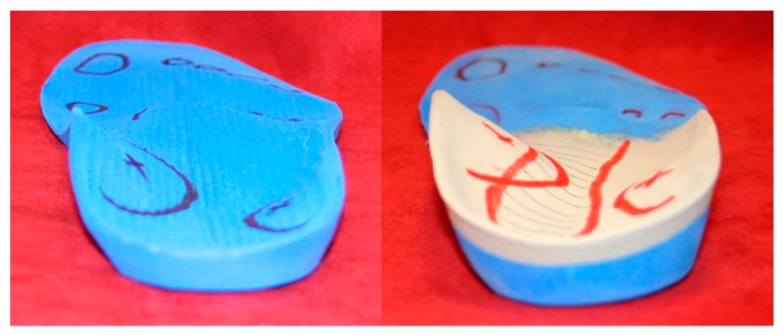

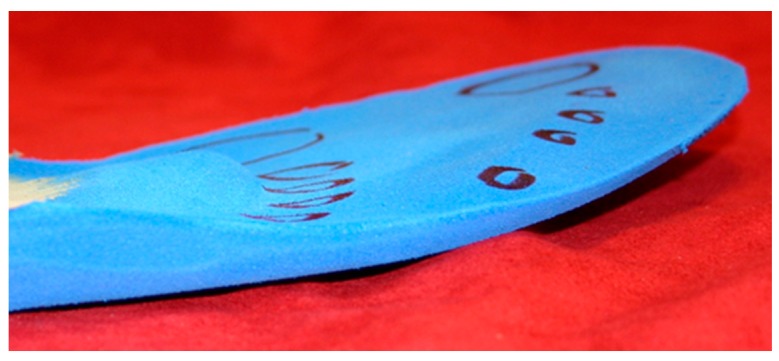
Customised foot orthoses.

In the foot orthoses group, the orthopaedic technician used blueprints and foot scanners as measuring instruments for the production of the orthoses. The orthoses were checked with pedobarography and medilogic soles (T&T MediLogic Medizintechnik GmbH, Schoenefeld, Germany). With the data obtained from the pressure distribution measurement, the orthoses (Footpower, FSGmbH, Gummersbach, Germany) were milled with three layers (shore hardness A 50, A 25 and A 35) and an additive support layer from ethylene vinyl acetate using computer-aided design and computer-aided manufacturing techniques. To relieve pressure of the origin of the plantar fascia, a canal (referring to the medial tuber calcanei) was milled and filled with soft material and a cushion layer was applied to the heel. Subsequently, the footprints were produced, and the orthoses were individually manufactured (Figure 2). The underlying idea of this type of foot orthoses is to relieve the plantar fascia, reduce heel pressure and pain and obtain a positive, non-restrictive effect on joint mobility without compromising the muscle activity of the foot itself. At the highest point of the foot orthoses, a medial support for the sustentaculum tali was moulded. Through raising the toe berries and using a retrocapital edged pelotte, pre-tensioning of the plantar fascia was expected.

### 2.4. Statistical Analysis

The AOFAS-AHS and FPF Scale data were transferred into Microsoft Excel and were then analysed with a statistical software package (IBM, SPSS version 23, Chicago, IL, USA). Descriptive data were presented as relative frequency, mean and standard deviation. Differences in the distributions were investigated by chi-square tests. Levene tests were applied to examine the variance homogeneity between the three groups. Differences in the changes in scale values from T0 to T3 between the three groups were investigated using an analysis of variance (ANOVA) and Bonferroni post hoc tests. Differences between T0 and T3 within each group were calculated using dependent Student’s t-tests. Differences in the numbers of physiotherapy and evaluation of the foot orthoses between the groups were investigated by independent Student’s t-tests. A *p*-value of ≤0.05 was assumed to be statistically significant.

## 3. Results

Sixty-three patients met the eligibility criteria. The mean age and duration of PF symptoms were 48.4 ± 9.8 years and 4.4 ± 1.3 months, respectively. There were no statistically significant differences for age, gender and body mass index between the three groups at T0 (*p* > 0.05). However, the patients of the FO group had statistically significant shorter duration of symptoms, fewer had back pain with shorter duration, lower FPFS values for work, and received fewer treatments and medications before starting the study than both other groups (Table 1).

A total of 58 (92%) patients appeared for the follow-up assessment after one month (T1). Five patients from the FO group were counted as drop-outs (Figure 1). A total of 47 (75%) patients completed the three-month follow-up (T0). Intention to treat was applied in the MT, FO and combined group for one (5%), six (29%) and four (19%) patients, respectively. There were statistically significant changes for the AOFAS-AHS (*p* < 0.01) and the FPFS (*p* < 0.01).

**Figure 3:**
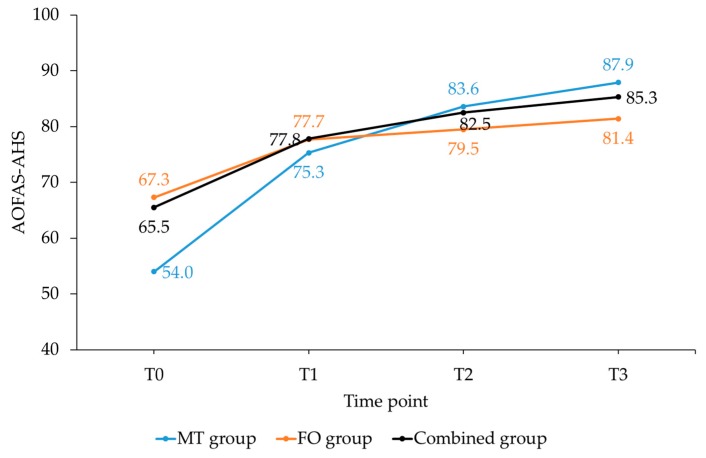
Changes in American Orthopaedic Foot and Ankle Score-Ankle Hindfoot Scale (AOFAS-AHS) for the three groups from T0–T3. Abbreviations: MT, manual therapy; FO, foot orthoses; T0, baseline; T1–T3, follow-up sessions after 1–3 months.

Between-group differences in the AOFAS-AHS and its subscales showed a greater improvement from T0–T3 in the MT group (*p* < 0.01) than the FO and combined group (Figure 3, Table 2).

**Figure 4:**
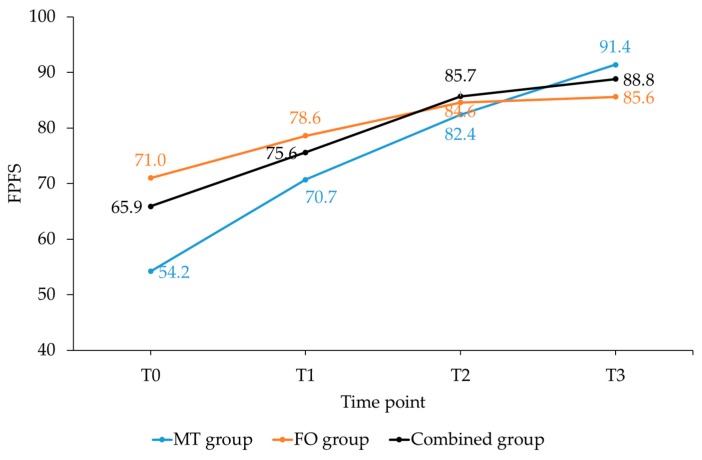
Changes in Foot Pain and Function Scale (FPFS) for the three groups from T0–T3. Abbreviations: MT, manual therapy; FO, foot orthoses; T0, baseline; T1–T3, follow-up sessions after 1–3 months.

Likewise, between-group differences in the FPFS and in its subscales showed that the MT group improved more from T0–T3 (*p* < 0.01) than the FO and combined group (Figure 4, Table 2).

Besides the statistically significant differences, all three groups showed clinically meaningful improvements over time. Differences in AHS from T0–T3 for the MT, FO and combined group were 35% (”much improved”), 15% (”minimally improved”) and 21% (”improved”), respectively. The corresponding FPFS changes were 37% (”much improved”), 18% (”minimally improved”) and 24% (”improved”), respectively. The FO and combined group did not reach the MCID in AHS subscale function. In all groups, the improvement in subscale pain was higher than in subscale function.

One FPFS question was ”first step” pain after a period of rest. The improvement of the values in the MT, FO and combined group were 50%, 32% and 43%, respectively. Effectiveness on pressure pain and weight bearing were similar; in the MT group for 56% and 59%, in the FO group for 41% and 41% and in the combined group for 45% and 42%. The values for pain during rest and while standing were lower, just as in the subscale function for weakness and stiffness. Restrictions in sports and work had an improvement of 51% and 30% in the MT group, 26% and 31% in the FO group and 28% and 20% in the combined group. The improvements in limping varied greatly between the groups; 41% in the MT group, 36% in the FO group and 16% in the combined group.

The number of treatments did not differ between the MT and combined group (*p* > 0.05).

## 4. Discussion

The results of our study showed that all three interventions for PF achieved both a statistically and clinically significant improvement over time. Furthermore, it suggests that manual therapy offers greater clinical benefits, reducing pain and improving function compared to customised foot orthoses and combined therapy. The application of manual techniques was standardised, and no additional forms of therapy were used, making the therapy reproducible and comparable to other studies with multiple concurrent therapies.

### 4.1. Comparison with Previous Studies

Cleland and colleagues [1] conducted a study in which 30 patients of a manual therapy group underwent a five minute aggressive soft tissue mobilisation directed at the triceps surae and insertion of the plantar fascia as well as a rear foot eversion mobilisation. It included an impairment-based manual therapy at the hip, knee, ankle and foot on the clinical decision making of the treating therapist. The other group with 30 patients was treated with electrophysical agents and exercises. Three outcome measures were reported: Lower Extremity Functional Scale (LEFS), Foot and Ankle Ability Measures (FAAM) and a numerical pain rating scale (NPRS). The overall group-by-time interaction showed significantly better results for the manual therapy group. In comparison to the Cleland study, the improvement reported in the pain subscale was greater at the three month follow-up than the six month follow-up, and the AHS and FPFS total scores were higher than in LEFS of Cleland et al. [1]. The underlying mechanism for improvements related to manual physical therapy in the study of Cleland et al. [1] could not be determined as a first level of standardised intervention was used in addition to a second level of intervention that utilised an impairments-based approach. Hence, it could not be determined with any certainty which specific manual therapy and exercise technique was most advantageous.

In our study, the focus was placed on treating local impairments of the joint structures of the foot and the proximal impairments of the spine. No stretching of the soleus and gastrocnemius muscle or plantar fascia took place, and no additional treatments of the knee and hip joints were performed. McClinton and colleagues [25] also reported an association between PF and lumbar back pain. The result of our study suggests that a therapy integrating the spine may help to alleviate the often long-lasting symptoms of PF and ultimately achieve a better result.

The Burmeister study [24] used a similar intervention methodology. In addition to the spine, a treatment of internal organs was conducted with 15 patients. Three osteopathic treatments were conducted within a three-week period. Despite improvement on some items, no significant difference could be detected between the verum and control group. It is possible that a higher number of treatments could have led to a further improvement; however, this was not tested.

In our study, many patients actually required three months of therapy with a mean of 10.6 treatments to be symptom free. Cleland et al. [1] also questioned whether more than six therapies would have resulted in a further improvement in function. The dosage also remained unclear in the included studies of the clinical practise guidelines of Martin and colleagues [13]. The majority of the studies selected by Mischke and colleagues in their review [4] evaluated the short-term effects of a treatment. This might be less meaningful in the often long-lasting course of PF.

Custom made foot orthoses versus a combined treatment of manipulation and mobilisation of the foot and stretching exercises with 10 patients per group were compared in a study by Dimou and colleagues [40]. A review by Hawke and colleagues [17] reported that both groups in the Dimou study had statistically significant reductions in pain on the NPRS. After only two weeks, there was a statistically significant difference in foot pain favouring mobilisation of the foot versus stretching, with no significant difference after one month and two months. The review indicated that the customised foot orthoses did not reduce foot pain more than non-customised or sham foot orthoses, including when combining them with stretching exercises or night splints. However, it is suggested that using customised foot orthoses and night splints together may reduce foot pain. The foot orthoses in our study complied with Hawke and colleague’s definition [17] of customised orthoses: fabricated according to practitioner-prescribed specifications, the orthoses should be contoured, removable in-shoe devices that are moulded or milled from an impression of the foot. In our study, customised foot orthoses were found to be less effective compared to manual therapy or combined treatment. The result also showed that wearing the foot orthoses over three months did not reduce the number of manual therapies required in comparison to the MT group.

Overall, PF still remains a “black box”. Besides insoles and manual therapy, different treatment options (e.g. resistance training, corticoid injections and extracorporeal shockwave therapy) seem to be reasonable in the treatment of PF. It is, however, unclear what the underlying mechanisms are. Additionally, it is important to highlight that individuals may respond differently to the various treatment options, meaning that there is no general or overall recommendation for the treatment of PF. In particular, the results of manual therapy for treating PF suggest that the pathophysiology seems to be more complex and not fully understood. Our study shows that joint mobility and low back pain play a role in treating PF. Joint dysfunction treated with manual therapy seems to lead to a functional improvement and a relief of symptoms. If these dysfunctions lead directly to altered mechanical loading of the plantar fascia or indirectly via the myofascial slings remains unclear. We found in our study that only a one- to two-week manual therapy intervention altered the symptoms of PF. It is unclear [41] if this easy-to-implement treatment can be used for preventive purposes, e.g. in athletes, as it requires more research.

### 4.2. Strength and Limitations

In this study, manual therapy and customised foot orthoses treatments were carried out in isolation, meaning that the methodology is reproducible, and any differences clearly assigned to the treatment condition. Based on experience from other studies in which 3–6 weeks of therapy were reported to be insufficient, the duration of treatment in our study was set at three months. Since the disease is often long-lasting with severe discomfort, patients should receive treatment in the control group, rather than to being exposed to placebo treatment over such a long period. The entire sample population of our study was recruited from one clinical practice, which could be seen as a possible limitation. As a limitation of our study, we did not perform a power analysis prior to the upcoming recruitment. However, it must be considered that we were the first to investigate the effectiveness of these three different treatment approaches in the management of PF within a randomised controlled trial and that we nevertheless found statistically significant differences in our data.

The baseline outcome scores of the foot orthoses group were significantly higher at the beginning of the study than those of the manual therapy and combined group. So, selection bias may have occurred. High scores at baseline generally complicate an improvement over the course of the study or even make it impossible. To enable an improvement, a limitation of the input values set on the scales in the inclusion criteria would have been useful. An attrition bias occurred, because participation in the follow-up assessment in the FO group with 10 patients, versus 20 in MT, respectively 17 in MT and FO (combined group) was significantly lower. To counteract possible bias, we performed an intention to treat analysis. The weekly treatment in the manual therapy group represented a more intense support for the patients over three months. In contrast, the patients of the foot orthoses group had one appointment with the orthopaedic technician for the footprints and a second to receive the fabricated foot orthoses. After receiving their orthoses, patients may have seen little reason for clinical follow-up appointments, resulting in a performance bias. This may have reduced the success rate, because a higher number of FO patients no longer participated in the evaluation compared to the manual therapy group.

The combination of manual therapy and customised foot orthoses should demonstrate whether two simultaneously applied interventions would improve the outcome more than one treatment in isolation. According to our study design, we intended to mobilise the local restrictions of the foot before producing the foot orthoses. This was however not possible in all cases. The actual procedure in the combination therapy could have contributed to the fact that the combined group hardly achieved any improvements in the items function and alignment of the foot axis, whereas in the manual therapy group, the results increased in both items. Some patients of the combined treatment group reported foot pain, possibly caused by the fact that the orthoses no longer fitted optimally after mobilisation of the restrictions of the feet. Therefore, differences were smaller in the manual therapy group than in the foot orthoses group. Caution should be applied when interpreting these results, as patients in the manual therapy group had significantly more complaints of accompanying back pain, which could be another explanation as to why manual therapy was more successful in this group.

### 4.3. Clinical Implications

A meaningful overall minimal clinically important change (MCID) could not be reported for NRS or AOFAS-AHS PHP (plantar heel pain) [36]. Additionally, it is a problem to calculate the mean difference in pain score for the treatment group and to compare it to the MCID, because MCID is a metric based on longitudinal differences in individuals and should be used in the same context [39]. Analyses of the relationships between changes in NPRS scores demonstrated a reduction of two points, or 30%, to be clinically important and were measured using a standard seven-point patient Global Impression of Change [37]. These results are similar to results that were found in lower back pain patients compared after physical therapy using a 15-point Global Rating of Change scale [42]. The Global Rating of Change scale has been criticised because it is a transitional scale that requires recall of prior health status [43]. The Global Rating of Change scale is not temporally stable, with a finding in one week not associating to functional results the following week. The Global Rating of Change scale is only correlated to functional measures up to three weeks [44], so it was not included as an additional measure in the current study.

## 5. Conclusions

Manual therapy, customised foot orthoses and the combined treatments achieved statistically and clinically significant improvements over time, with the greatest effect for the treatment of PF being found in the manual therapy group. In addition, the results indicate that integrating spinal treatment for patients experiencing back complaints together with PF could improve treatment outcome.

## Figures and Tables

**Table 1:** Demographic data, symptoms, and therapies of the three groups at T0.

Variable	MT Group (n = 21)	FO Group (n = 21)	Combined Group (n = 21)	*p*-Value
Female sex, n (%)	16 (76.2)	14 (66.7)	14 (66.7)	0.74**^1^**
BMI (kg/m**^2^**)	28.3 ± 6.2	30.4 ± 4.8	29.4 ± 4.2	0.43**^2^**
Duration of PF symptoms (month)	5.3 ± 0.8	2.9 ± 1.8	5.0 ± 1.2	<0.01**^2^**
Back pain, n (%)	19 (90.5)	6 (28.6)	17 (81.0)	<0.01**^2^**
Duration of back pain (years)	11.0 ± 8.1	2.5 ± 6.0	9.0 ± 8.6	<0.01**^2^**
Work: Standing, weight bearing, n (%)	11 (52.4)	7 (43.7)	13 (61.9)	0.02**^2^**
Sporting activities, n (%)	17 (81.0)	12 (57.1)	18 (85.7)	0.08**^2^**
Therapy pre-study, n (%)	17 (81.0)	8 (38.1)	17 (81.0)	<0.01**^1^**
Duration of therapy pre-study (month)	3.4 ± 2.4	1.2 ± 2.1	3.5 ± 2.4	<0.01**^2^**
Medications at the start of the study, n (%)	12 (57.1)	6 (28.6)	14 (66.7)	0.02**^1^**

Abbreviations: MT, manual therapy; FO, foot orthoses; BMI, body mass index; n, number; PF, plantar fasciitis; **^1^** Chi square tests; **^2^** ANOVA.

**Table 2:** Comparison of the mean improvements in American Orthopaedic Foot and Ankle Score-Ankle Hindfoot Scale (AOFAS-AHS), Foot Pain and Function Scale (FPFS) and their subscales for the three groups from T0–T3.

Variable	MT Group (n = 21)	FO Group (n = 16)	Combined Group (n = 21)
AOFAS-AHS	33.9*	14.1	19.1
Pain subscale	48.8*	26.3	26.3
Function subscale	24.5*	6.1	6.1
FPFS	37.2*	14.6	22.9
Pain subscale	48.4*	28.4	28.4
Function subscale	32.8*	19.0	19.0

Abbreviations: MT, manual therapy; FO, foot orthoses; * Statistically significant higher (*p* < 0.01) than in the other groups.
